# Glandular odontogenic cyst: an uncommon entity

**DOI:** 10.1016/S1808-8694(15)30800-4

**Published:** 2015-10-19

**Authors:** Michelle Manzini, Christian Deon, Liliam Dalla Corte, José Carlos Bertotto, Luciana Boff de Abreu

**Affiliations:** 1 Fourth year medical student, medical course of the Universidade de Caxias do Sul; 2 Fourth year medical student, medical course of the Universidade de Caxias do Sul; 3 Fourth year medical student, medical course of the Universidade de Caxias do Sul; 4 Full professor and coordinator of the Otorhinolaryngology Discipline (Unidade de Ensino Médico Otorrino-Oftalmo) medical course of the Universidade de Caxias do Sul; 5 Fourth year medical student, medical course of the Universidade de Caxias do Sul. Universidade de Caxias do Sul

**Keywords:** bone cysts, jaw neoplasms, tumor

## INTRODUCTION

The glandular odontogenic cyst is a cyst that arises in development sites of the teeth; it is usually found in the mandible and maxilla,1 and is characterized by cuboidal or columnar epithelium on the surface and in crypts within the epithelium.

This cyst generally is encountered in the anterior areas of the mandible; it is more common in adults aged over 40 years, and tends to recur. The recorded prevalence ranges from 0.012 to 1.3%.^2^

It is a slow-growing cyst that affects both sexes similarly.^2^

It is a relatively new entity that was first described in 1987, and recognized by the World Health Organization in 1992. Because of the paucity of reported cases, there is no consensus or established protocols about many aspects of management of this lesion.

## CASE REPORT

CRVM, a white female patient aged 27 years, residing in Caxias do Sul, self-employed, with a history of smoking, sought an outpatient clinic (Ambulatorio Central) complaining of pain and pruritus on the left maxilla, difficulty to eat, and halitosis. She reported fruitless previous treatments with amoxicillin and diclofenac. The physical examination showed edema and hyperemia in the anterior portion of the left maxilla.

Laboratory work-up consisted of liver function tests, serum iron and preoperative tests, all of which were within normal limits.

A plain radiograph of the face revealed a large bony lesion in the left maxilla.

**Figure 1:**
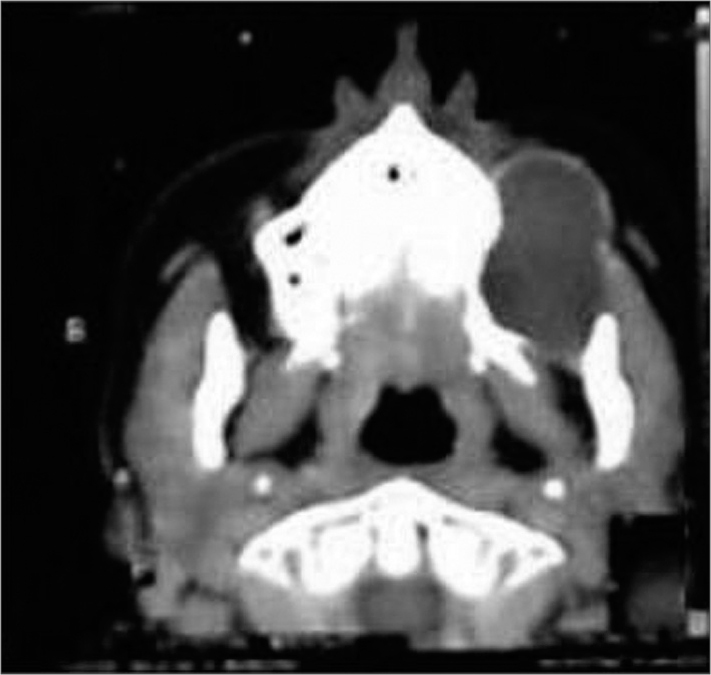
Computed tomography showing an intraosseous cyst in the left maxilla.

Computed tomography of the middle third of the face showed an osseous cyst with dense content, measuring about 6.0 cm along its longest dimension, located deep in the left maxillary sinus next to the lateral wall of the left maxillary bone; the dental alveoli were involved (Fig. 1).

The patient underwent surgery, which consisted of enucleation of the maxillary cyst. Macroscopically it was a grayish, elastic tissue measuring 2.0 × 1.0 × 0.3 cm. Its liquid content was aspirated and the surrounding capsule was removed; both were sent to histology and pathology for analysis. The pathological diagnosis was glandular odontogenic cyst.

The patient recovered uneventfully in the immediate postoperative period.

## DISCUSSION

The main clinical finding in this disease is painless local edema; the clinical picture, however, is non-specific.^3^ The lesion may cause pain due to compression of a neurovascular bundle or secondary infection; inflammation, however, is uncommon. There may also be paresthesia, depending on the site of the lesion, or a feeling of pressure on the dental arcade.

This cyst develops in the maxilla1 or in any area of the mandible, mostly in the anterior portion. It may mimic, on the surface, the central mucoepidermoid carcinoma.^4^ It is generally located inside bone, and may appear as a unilocular or multilocular lesion in radiographs.

Lack of consistent clinical manifestations and the intraosseous development of these lesions mean that radiography is essential. Radiographic findings include a rounded or oval lesion, usually with well-defined borders.

Most authors agree that there are no radiographic features specific of glandular odontogenic cysts. The differential diagnosis is made with botryoid cysts, keratocysts, residual cysts, the central mucoepidermoid carcinoma, and the ameloblastoma.

A malignant potential for this lesion has been suggested in the medical literature; expansion and perforation occur in many of these cases. Computed tomography is indicated in cases with large multilocular lesions involving the facial sinuses, floor of the nose and orbit.

Histologically it is a polycystic structure with a non-keratinized squamous epithelium and cuboidal or ciliated epithelium with mucus-secreting cells.

There is histological similarity with the central mucoepidermoid carcinoma.^4^ Certain authors have suggested using immunohistochemical markers to differentiate these tumors.^2^

Treatment options include curettage, enucleation, and en bloc local excision. Some authors recommend en bloc local excision with primary repair, which aims to cure the patient and avoid further surgery, since there is a high recurrence rate with the conservative treatment, and the lesion may become invasive.^5^ Others recommend conservative procedures with postoperative follow-up during 3 to 5 years.^3^

## FINAL COMMENTS

Although this is an uncommon disease that has been recognized only recently, and that opinions diverge about the proper strategy, this disease should be included in the differential diagnosis of mandibular or maxillary lesions that are loculated and radiolucent in radiographs.
